# Display in the Wild (DIW): An Adaptive Projection-Imaging System to Screen Geometry in Real Time

**DOI:** 10.3390/s18093063

**Published:** 2018-09-12

**Authors:** Byungyong Ryu, Sung-Ho Bae

**Affiliations:** Department of Computer Science and Engineering, Kyung-Hee University, Gyeonggi-do 17104, Korea; read100nm@khu.ac.kr

**Keywords:** TV, monitor, projector distortion, distortion correction, calibration, depth camera, Unmanned Aerial Vehicles, UAVs, display

## Abstract

TVs and monitors are among the most widely used displays in various environments. However, they have limitations in their physical display conditions, such as a fixed size/position and a rigid/flat space. In this paper, we suggest a new “Display in the Wild” (DIW) concept to overcome the aforementioned problems. Our proposed DIW system allows us to display a flexibly large screen on dynamic non-planar surfaces at an arbitrary display position. To implement our DIW concept practically, we choose a projector as the hardware configuration in order to generate a screen anywhere with different sizes. However, distortion occurs when the projector displays content on a surface that is dynamic and/or non-planar. Therefore, we propose a distortion correction method for DIW to overcome the aforementioned surface constraints. Since projectors are not capture devices, we propose using a depth camera to determine the distortions on the surfaces quickly. We also propose DIW-specific calibration and fast/precise correction methods. Our calibration method is constructed to detect the projection surface easily and quickly, and also allows our proposed system to accommodate the intrinsic parameters such as a display resolution and field of view. We accomplish a fast undistortion process of the projector by considering only surface boundary pixels, which enables our method to run in real time. In our comprehensive experiments, the proposed DIW system generates undistorted screens such as TVs and monitors on dynamic non-planar surfaces at an arbitrary display position with Unmanned Aerial Vehicles (UAVs) in a fast and accurate manner, demonstrating its usefulness in practical DIW scenarios.

## 1. Introduction

TVs and monitors are among the most widely used display devices in the industry and at home. These display devices have evolved to larger screens and thinner thicknesses [1], but are still fundamentally built-in displays with rectangular screen shapes. Such conventional display devices have various fundamental constraints. First, they require as much flat space as the device size. Second, the space in which the display is located should be invariant. Third, the screen size is limited by the size of the device, resulting in less flexibility. Fourth, they work only in the installed space [2].

If there is a new display that can produce a TV or monitor screen on a dynamic non-flat surface that is flexible in terms of both screen location and size, it can overcome the aforementioned constraints of conventional display devices. We describe such a new device as “Display in the Wild” (DIW), and this device will be applicable and valuable in various industries. For example, to display a large screen at outdoor performances or events, we typically need to install a large flat screen or connect numerous smaller TVs. With DIW, on the other hand, we can use Unmanned Aerial Vehicles (UAVs) and a lightweight canvas to quickly configure a large screen at a low cost and display a TV-like screen on a dynamic non-planar surface. DIW can also create a TV-like screen on curtains that are not flat while being easily found at home, as well as on tents that are commonly used in outdoor camping.

In this paper, we propose both hardware and software configurations to implement DIW. For the hardware, we choose a projector as a suitable display device to overcome the limitations of fixed positions and sizes, as projectors can generate a variable-sized screen anywhere. However, geometric distortions can occur when the projector displays on a surface that is dynamic and non-planar. To solve this problem, a geometric distortion correction method is required to display an undistorted screen (e.g., the rectangular screen on a TV). Here, a capture device that can predict the distortion is needed since the projector itself cannot sense the screen geometry. Given that a depth camera can quickly capture 3D surfaces in real time, we decide to use one as a capture device to predict the distortion on dynamic non-plane surfaces. In summary, we utilize a projector and a depth camera for our hardware configuration to implement DIW.

In order to complete the DIW system with the proposed hardware configuration, we developed essential software configurations for DIW: (i) a quick prediction method for geometric distortion on dynamic non-planar surfaces, and (ii) a fast correction method for geometric distortions. Although there are many distortion-correction methods for projection, the existing methods hardly correct the distortions present in dynamic non-planar surfaces. In fact, they mainly project the structure patterns on surfaces to extract and match features using RGB cameras and find distortions in a reconstructed 3D space [3,4,5,6,7,8,9]. However, it is difficult to cope with dynamic distortion because the projector may generate patterns that interfere with viewing of the screen contents. Some methods [10,11,12,13] can deal with dynamic distortion by projecting non-visible patterns. However, such methods also have drawbacks, in that they often unreliably extract and match features in certain lighting environments, thus failing to correct the distortions. Moreover, the existing feature extraction and matching processes usually require heavy computation, which makes them inappropriate for predicting dynamic distortions at high speed. In addition, to fill in missing information on the surface, they fit 3D mesh shapes to the distorted surface in the correction step [7,9], which also results in a high computation complexity. Therefore, the conventional projector correction methods are hardly applicable to DIW systems, which require fast and reliable correction methods on dynamic non-planar surfaces.

Additionally, most correction-methods perform calibrations between the projector and capture device. Some existing manual and/or automatic calibration methods have shown good results with RGB or depth cameras [14,15,16,17,18,19,20,21,22]. However, existing calibration methods are not suitable for DIW, since they focus only on the accuracy of calibration between the projector and capture device, rather than fast detection of the geometric distortion of the projection. Furthermore, some calibration methods may need to re-calibrate when intrinsic parameters change (e.g., display resolutions) in the projector, as the method involve intrinsic parameters in the calibration process.

**Figure 1:**
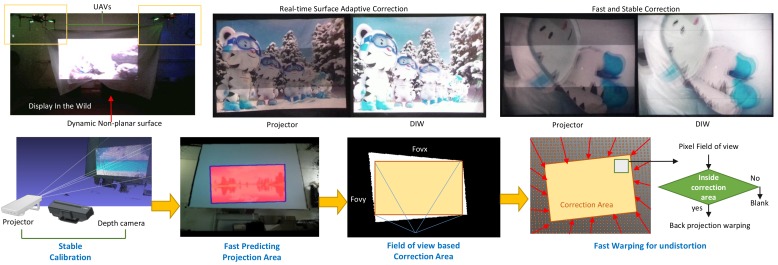
A practical example of our DIW system. We made a screen in the wild having dynamic and non-planar characteristics, using UAVs and a lightweight canvas. Our system can quickly predict/correct the geometric distortions of the projector.

Therefore, to overcome the aforementioned problems of the existing methods and complete the DIW system with the proposed hardware, we propose: (i) a quick estimation method for geometric distortions from the projection; (ii) a fast distortion correction method; and (iii) a DIW-specific calibration between the projector and depth camera. Using the depth camera, our method can quickly predict/correct distortions on the projection surface. This is because we specialize our calibration method for fast detection of the projection surface in the depth image. To efficiently find the correction area of the user’s viewpoint, only the boundary pixels on the detected projection surface in the depth image are used. When determining warping and correction, the proposed method can quickly recover the high-resolution depth map of the projection surface by adopting a Gaussian weighted interpolation process that fills out the missing information in the original depth map for a very low computation cost. This method can provide an alternative to the traditional mesh fitting methods that result in high computation costs. In addition, the proposed calibration method focuses on extrinsic parameters (i.e., the transformation matrix for positional relationships between the projector and depth camera), making it easy to respond to display resolution changes. Figure 1 illustrates the overall process of our proposed DIW system. The main contributions of our work are summarized as follows:

- We first devise the concept of “Display in the Wild” as a new display device that overcomes the limitations of fixedness for both display position and surface condition that are present in existing display devices.
- We propose a new hardware configuration specialized for DIW that couples a projector with a depth camera for fast and reliable corrections to the distortions that can occur due to dynamic non-planar surfaces.
- To complete this DIW system, we also propose software configurations that allow the DIW display to be deployed in real time applications. Our software configurations include a fast geometric distortion estimation, correction, and DIW-specific calibration. These allow us to perform robust projector correction without using markers.
- We prove the usefulness of our DIW system by performing comprehensive quantitative and qualitative tests under dynamic non-planar surface conditions.

This paper is organized as follows: In Section 2 we discuss the existing methods for distortion correction of the projector and for calibrations. Then, in Section 3 we describe our fast correction and calibration methods for the DIW system. We thoroughly conducted quantitative and qualitative experiments to demonstrate the performance of the proposed method, as described in Section 4. Lastly, we discuss limitations and conclusions for our proposed methods in Section 5 and Section 6, respectively.

## 2. Related Work

### 2.1. Correction of Projection Distortion

The projector can provide a large display screen at a relatively low cost but has a fatal disadvantage in that projection distortions can easily occur depending on the surface geometry and projection angle. When an image is projected on a flat surface without appropriate projection angles, a trapezoid-like distortion is caused, which is called the keystone effect. This problem can be corrected by adjusting the projector’s position manually or performing 2D projective transformations on an image. In this regard, many correction methods have been proposed to eliminate keystone distortions [23,24,25,26]. These methods slightly differ in the details, but mainly calculate the homography between the projected and original images using RGB cameras.

Besides this, when using the projector irregular distortions can occur when projecting an image on a non-planar surface. Such distortions cannot be corrected by a homography transformation as in the keystone case. Instead, predicting the irregular geometry of the surface is essential for correction. To correct distortion produced by specific non-planar surfaces, [27,28,29,30,31] proposed parametric surface models, such as cylindrical, conic, dome, and intersecting planar models. These methods can correct distortion on a non-planar surface from specific shapes, but it is difficult to apply them to non-planar surface shapes outside typical parametric surface models.

It is worth noting that our DIW system aims at automatic projector correction on a non-planar surface, which is different from projector deformation methods [32,33,34,35]. The main difference is that the projector deformation methods aim at texture mapping to deformable objects for Augmented Reality, involving some computer vision algorithms such as object segmentation/detection and template-based 3D shape mapping [32,35]. To overcome inaccurate behaviors of these computer vision algorithms, many recent projector deformation methods rely on adopting markers to easily find target objects and warping points [33,34].

On the other hand, our method belongs to the automatic projector correction methods where neither markers nor sophisticated computer vision algorithms are needed. The proposed methods aim to minimize distortions in a display caused by non-planarity of a target surface and projector geometry.

To correct geometric distortions on arbitrary surfaces, approaches based on structured light patterns have been proposed [3,4,5,6,7,8,9]. These methods project structured light pattern images on non-planar surfaces and captured the projected patterns by using RGB cameras. With these structured light patterns, 3D points on the projection surface can be calculated, and the correction can be performed according to the 3D shapes on the surface. However, when the shape of the projection surface changes, the shape information for the projection surface needs to be updated. For this, the projector needs to continually project the structured patterns onto the screen, which creates discontinuity in the displayed contents that the users are watching. This limitation makes it difficult to correct distortions that occur due dynamic non-planar surfaces, which can be solved via DIW systems.

To solve the updating problem of dynamic non-planar surfaces, imperceptible pattern-based correction methods have been proposed [10,11,12]. They calculate the 3D points of the distortion surface by inserting imperceptible patterns onto the projection image, which are unnoticeable to the users watching the display. However, they require a high computation power for estimating the model parameters of the dynamic non-planar surfaces, which often change in real time. On the other hand, Setkov et al. corrected the image geometry by using color invariant features [36]. This method can correct the dynamic distortion without beaming structured light patterns. However, it is difficult to apply this method to irregular surfaces since it adopts a global homography that limits reflections of the geometry for non-planar surfaces. Ito et al. proposed a stereo vision-based correction method to capture the geometry of a surface without disturbance for the structured light patterns on the screen [13]. This method estimates a perspective projection matrix using SIFT (Scale-Invariant Feature Transform) [37] and obtains the 3D shape of a non-planar surface using POC (Phase-Only Correlation) [38,39,40,41]. Therefore, Ito’s method is able to continuously predict and correct the distortion resulting from dynamic projection surfaces. As described above, many methods have been proposed for correcting the distortion, but only some of them can be applied to dynamic non-planar surfaces, which is thus a desirable functionality for our DIW system.

Although most of the existing methods use RGB cameras to calculate the 3D shape of the surface, they have intrinsic drawbacks when applied in our DIW scenario. First, to correct the distortion for a rapidly varying non-planar surface, it is essential to develop a fast 3D shape acquisition method for the real-time DIW system. However, to calculate the 3D shape of the surface, RGB camera-based correction methods inevitably perform feature-detection/matching processes. In this case, when the image resolutions are sufficiently high, such as HD (720×480) and FullHD (1920×1080), the traditional feature-detection/matching processes fail to run in real time [42]. Second, the projection area may not have sufficient contrast to detect the feature point, as projectors are mostly used in dark environments that make the captured images in the projection area relatively brighter and saturated. These two problems cannot be solved using the traditional correction methods with RGB-cameras. Instead of using an RGB camera, we incorporate a depth camera into the projection-based DIW system to correct distortions on the dynamic non-planar surfaces under real-time and practical dark ambient illumination conditions.

### 2.2. Camera to Projector Calibration

Calibrating the positional relationship between the camera and projector is an essential part of predicting the projection area in 3D coordinates. RGB cameras are unable to capture 3D geometry information directly, so, existing calibration methods use to beam specific patterns on a surface that the RGB camera can capture, thus estimating the 3D geometry. To obtain a higher accuracy, multiple sets of features are often extracted from the calibration patterns at different orientations. Then, the world coordinates of the features are estimated with respect to minimizing the reprojection errors. Most calibration methods between RGB cameras and projectors [14,15,16,17,43] perform in a similar manner to that described above.

On the other hand, some calibration methods utilize a depth camera since 3D points can directly and easily be calculated via depth images [20,21,22]. However, existing calibration methods with a depth camera are not specialized for a fast prediction on the projection surface, but rather accuracy of the calibration parameters. Furthermore, some of these methods require re-calibration when the resolution (i.e., the intrinsic parameters of the projector) changes, which often occurs in display devices. Therefore, we propose a simple calibration process to adapt a depth camera as our hardware configuration for DIW, which will be fully described in Section 3.2.

## 3. Proposed Method

In this study, we developed our DIW system based on the assumptions that: (i) the projector and depth camera are attached and fixed together; (ii) that the target surface projected by the projector is non-planar and dynamic; and (iii) that the depth camera is placed parallel to the longitudinal axis. Our proposed DIW system calculates the relationship between the projector and depth camera (the extrinsic parameters) at first using our proposed calibration method. After that, the projector distortion is corrected in real time through the following iterative process:

1. A target surface area that is projected by the projector is predicted quickly via the depth image with the estimated calibration parameters.
2. The most effective correction area (i.e., the maximum rectangular region inside the projection surface) is calculated for the undistortion processing on the display.
3. Finally, the original projection image is warped into the effective correction area, and the projector outputs the warped image in order to remove the geometric distortion on the target surface.

### 3.1. Hardware Configuration for the DIW System

We use a projector and depth camera as the hardware configuration to implement our DIW system. In this paper, we choose Kinect v2 and a portable laser projector (Model: Celluon PicoBit B06X93WFBP) where Kinect v2 captures depth images with 30 frames per second (fps) and the projector displays a screen with 60 Hz refresh rates. In choosing hardware configurations, we consider the following important conditions.

First, Kinect v2 has already demonstrated its performance in sensing precise depth images in many fields and has easy development. Even though it has a somewhat larger size than the recent Intel RealSense Depth Camera D435, we determined that the size of Kinect v2 is not big enough to interfere with the mobility, and further Kinect v2 offers fast implementation and testing advantages. Second, we use a laser projector instead of any existing LCD (Liquid-Crystal Display) or DLP (Digital Light Processing) projectors. These conventional projectors have a disadvantage in that one must adjust the focus according to the projection distance from lens. Because a DIW system should be able to generate the screen on a dynamic non-planar surface, adjusting the focus can be a complex problem for correcting the distortion in a DIW display. Moreover, in general LCD or DLP projectors have a large size. This creates a problem in mobility for easily creating a screen at a desired position. Unlike conventional projectors, the laser projector does not use a lens, so we do not need to adjust the focus. Furthermore, the laser projector is very small, so we decided it was suitable for the DIW system.

It is worth noting that, the physical allowable rotation angle and distance between the projector and the screen are limited in hardware configurations. Physically, the allowable rotation angle is dependent on the vertical/horizontal FOV(field of View)s of the projector. For example, when the vertical/horizontal FOVs are $Fov_{y}$ and $Fov_{x}$, the allowable angular range for vertical direction is $\pm \frac{Fov_{y}}{2}$ and for horizontal $\pm \frac{Fov_{x}}{2}$. Also, the allowable distance of the DIW system can be determined by the projection limit of the projector and sensing limit of the depth camera. Usually Kinect v2 has an allowable distance of eight meters, and the projector has a longer allowable distance, so eight meters can be judged as the allowable distance of our DIW system.

### 3.2. Projector to Depth Camera Calibration

**Figure 2:**
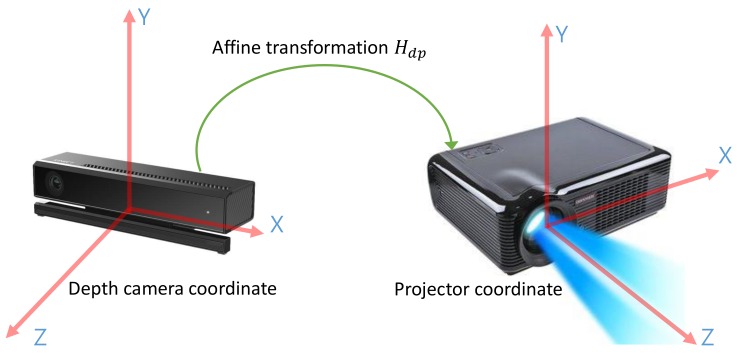
An example of the relation between the depth camera and projector coordinates, where the affine transform matrix $M_{dp}$ transforms the depth camera coordinates ($P_{d}$) into the projector coordinates ($P_{p}$).

We first calibrate the projector and depth camera in closed form. For this, we denote the 3D depth camera coordinates centered at the depth-camera position with points $P_{d}={\left(x_{d},y_{d},z_{d},1\right)}^{T}\in R^{3}$, and the 3D projector coordinates centered at the projector position with points $P_{p}={\left(x_{p},y_{p},z_{p},1\right)}^{T}\in R^{3}$, as shown in Figure 2. The affine transform matrix $M_{dp}$ that transforms the $P_{d}$ into $P_{p}$ is defined as

(1) $$P_{p}=M_{dp}P_{d},\;M_{dp}=\left(\begin{array}{cc} R_{dp} & T_{dp}\\ 0 & 1 \end{array}\right),$$

where $R_{dp}\in R^{3\times 3}$ and $T_{dp}\in R^{3}$ represent the rotation (consisting of $r_{11},r_{12},\cdots ,\;r_{33}$) and translation (consisting of $t_{1},t_{2},t_{3}$) matrices in the affine transform $M_{dp}$, respectively.

We can easily obtain $P_{d}$ using the depth image and its intrinsic parameters. On the other hand, if $P_{p}$ is not directly available, the projector does not capture any geometric information. Instead of $P_{p}$, we use 2D projection image coordinates for the images projected onto the target surface. We denote the 2D projection image coordinates as $P_{i}=\left(x_{i},y_{i}\right)$, which corresponds to $P_{p}$. The mathematical representation for the mapping of $P_{p}$ to $P_{i}$ is represented as:

(2) $$\begin{array}{c} x_{i}=f_{px}x_{p}z_{p}^{-1}\\ y_{i}=f_{py}y_{p}z_{p}^{-1}, \end{array}$$

where $f_{px}$ and $f_{py}$ are the width and height focal lengths of the projector, respectively. The above transformation $P_{p}=M_{dp}P_{d}$ can be expressed using the 2D projection image coordinate $P_{i}$ as:

(3) $$\begin{array}{c} \frac{x_{i}}{f_{px}}=\frac{r_{11}x_{d}+r_{12}y_{d}+r_{13}z_{d}+t_{1}}{r_{31}x_{d}+r_{32}y_{d}+r_{33}z_{d}+t_{3}}\\ \frac{x_{i}}{f_{px}}=\frac{r_{21}x_{d}+r_{22}y_{d}+r_{23}z_{d}+t_{2}}{r_{31}x_{d}+r_{32}y_{d}+r_{33}z_{d}+t_{3}}. \end{array}$$

To calculate elements of the transformation $M_{dp}$ in Equation (3) for more than one pair of $P_{i}$ and $P_{d}$, we form a linear system Ax=b, the details of which will be described in Appendix A. Then, by gathering the pairs, we solve the linear system using an SVD solver via our calibration process. It is worth noting that the SVD solver is known to yield a stable solution for a linear equation Ax=b.

**Figure 3:**
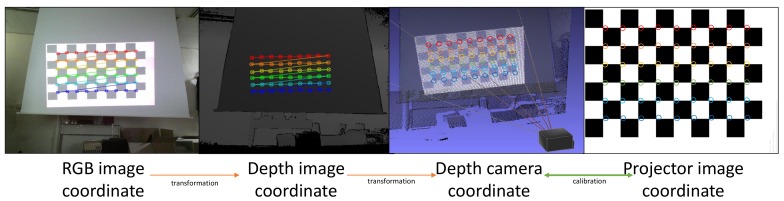
An example of the sampling procedure for calibration between the projector image coordinates and the depth camera coordinates.

The proposed DIW system projects a chessboard pattern onto a surface using the projector and captures the projected chessboard pattern using the RGB camera embedded in the depth-camera to gather the pairs. Note that most depth-cameras contain built-in RGB cameras for sensing color information. Then, our system detects the corners of the projected chessboard pattern using the captured image by implementing a corner detection method in the OpenCV library [44]. We map the chess board corners that are detected via the RGB camera to the corresponding positions in the depth image using the calibration values of the depth camera, which are provided by the manufacturer. Figure 3 shows the overall process of gathering 3D points at the depth camera coordinates and the corresponding 2D coordinates at the projector image.

**Figure 4:**
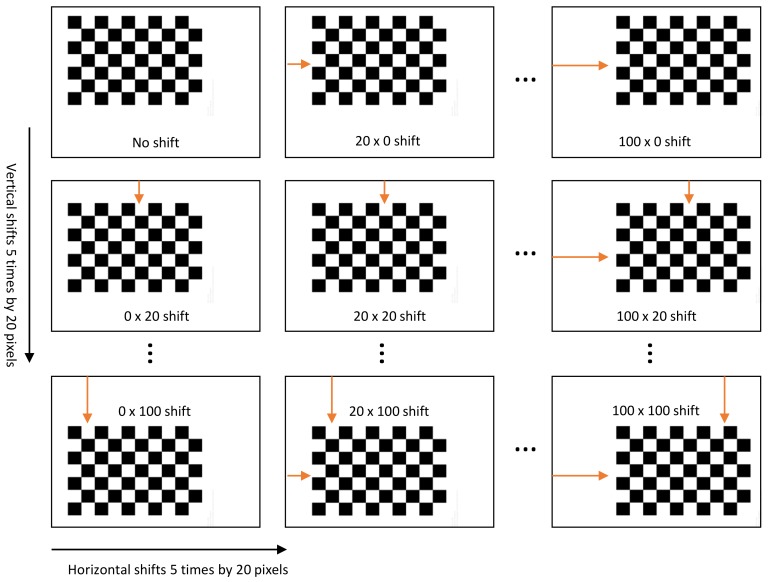
An example of generating chessboard patterns with 25 different positions. Our calibration system projects the chessboard pattern by moving 5 times by 20 pixels in both the horizontal and vertical directions so that a total of 1350 samples can be gathered in this process.

To collect a large number of sample points, our method generates a chessboard pattern with 25 different positions, moving the chessboard pattern 5 times by 20 pixels in both the horizontal and vertical directions. The chessboard that we used in this study has 54 corners (9×6) which can be obtained per each chessboard image. Therefore, the total 1350 corners can be gathered with 25 different images as shown in Figure 4. This calibration with a large number of sample points yields an accurate performances, which is given in Section 4.

The proposed calibration method can be used directly to detect the projection surface of the projector since it calculates the relationship between $P_{d}$ and $P_{i}$ via Equation (3). In addition, since the intrinsic parameters of both the depth camera and the projector are not included as unknown parameters in our calibration, it is possible to easily cope with a change in the projector resolution.

### 3.3. Projection Surface Prediction

Once we obtain the transformation matrix $M_{dp}$ through the proposed calibration, we can calculate the $P_{i}$ corresponding to $P_{d}$.

**Figure 5:**
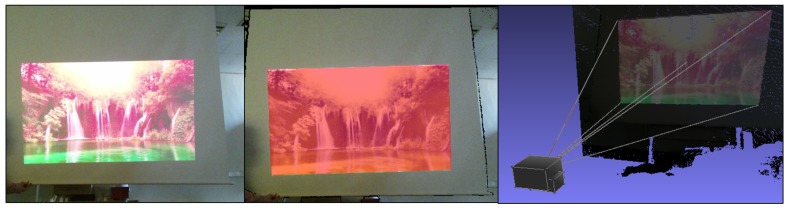
An example of the projection surface masks $M_{s}\left(x,y\right)$ obtained by Equation (4). The left image is a captured image with the projection and its background. The red region of the center image represents the projection surface masks. The right image shows the projection surface masks (colored regions) in 3D projector coordinates with five projection rays (white lines) at the boundaries and center.

We convert the depth image $I_{d}\left(x,y\right)$, where (x,y) represents the pixel position, into 3D point clouds $P_{d}\left(x,y\right)$ at the 3D depth camera coordinates using the predefined intrinsic parameters. Substituting each 3D point of $P_{d}\left(x,y\right)$ into Equation (3), we can calculate the corresponding 2D projection image coordinates $P_{i}\left(x,y\right)$. We then propose using the projection surface masks $M_{s}\left(x,y\right)$ for the depth image by using the simple and cost-effective equations as follows:

(4) $$M_{s}\left(x,y\right)=\left\{\begin{array}{cc} 255 & \mathrm{if}\;0\;\leq \;x_{i}\;<w\;\;\;\mathrm{and}\;\;\;0\;\leq \;y_{i}\;<h,\;x_{i},\;y_{i}\in P_{i}\left(x,y\right)\\ 0 & \mathrm{otherwise}, \end{array}\right.$$

where *w* and *h* are the width and height of the 2D projection image, respectively, and $x_{i}$ and $y_{i}$ are the pixel positions at $P_{i}\left(x,y\right)$. If the projection surface masks $M_{s}\left(x,y\right)$ is 255, the pixel of the depth image at (x,y) belongs to the projection surface as shown in Figure 5. In this way, we can simply detect projection regions based on the depth image in real time.

### 3.4. User Position

In this paper, we correct the distortion of the projection based on the assumptions that: (i) the user looks at the center of the screen (projection surface); and (ii) the user’s position is already known. In order to consider the user’s viewpoint in the correction process, we define 3D user coordinates with the user’s center as the origin, viewing this direction as the *z*-axis, left-to-right as the *x*-axis, and bottom-to-top as the *y*-axis. The user coordinates are defined relative to those of the projector one for easy adaptation of warping-based correction, which is fully described in Section 3.6. We also denote a point in the user coordinates as $P_{u}={\left(x_{u},y_{u},z_{u},1\right)}^{T}\in R^{3}$. Finally, the affine transform $M_{pu}$ that transforms $P_{p}$ into $P_{u}$ is defined as

(5) $$P_{u}=M_{pu}P_{p}=M_{pu}M_{dp}P_{d},\;M_{pu}=\left(\begin{array}{cc} R_{pu} & t_{pu}\\ 0 & 1 \end{array}\right),$$

where $R_{pu}\in R^{3\times 3}$ and $T_{pu}\in R^{3}$ represent the rotation (consisting of $r_{11},r_{12},\cdots ,r_{33}$) and translation (consisting of $t_{1},t_{2},t_{3}$) matrices in the affine transform $M_{pu}$, respectively.

From our experiments, we determined that the slight variations in the user position and viewpoint produce perceptually unnoticeable geometric distortions on the screen. This is because of the auto-compensation mechanism of the human visual system (HVS) [45], that is, the distortions produced by viewpoints are not noticeable even if the position of the user system is roughly approximated.

Note that this paper focuses on flexible and fast corrections with a projector and a depth-camera for the DIW system. Estimating a user’s position with a variety of poses and occlusions requires advanced user-detection algorithms with very high computation costs [46,47]. Most methods detect the user’s position in standing conditions, but, this is outside the scope of our paper. Therefore, we manually set the user’s position so that our DIW system runs in real time.

### 3.5. Correction Area Calculation

**Figure 6:**
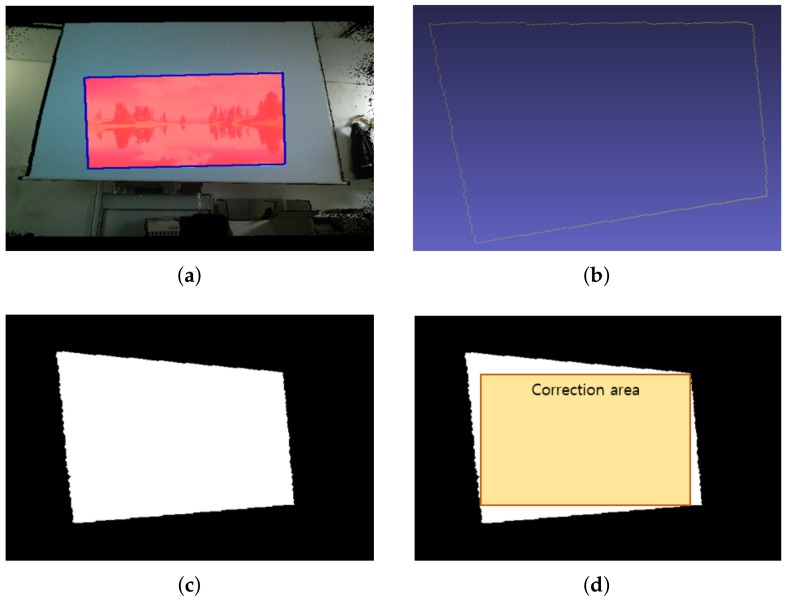
An example of maximum rectangular region detection for the correction area: (**a**) the detected boundary on the projection surface masks depth image $M_{s}\left(x,y\right)$; (**b**) 3D points of the detected boundary at the 3D user coordinates; (**c**) the rasterized image of the projection area; (**d**) the final maximum rectangular region calculated from the correction area.

To correct the geometric distortion on the projection surface, it is necessary to determine the correction area that shows the maximal rectangular image region to the user. In this study, we argue that it is inefficient to calculate the correction area using all of the 3D points that belong to the projection area. Instead, we propose calculating the correction area using only the boundary pixels at the projection area, which is simple and efficient, as shown in Figure 6.

First, we find the boundary pixels of the projection area on the projection surface masks $M_{s}\left(x,y\right)$ using the fast contour detection method [48]. When several contours are detected due to discontinuities of the projection surface, we choose the largest contour as the boundary to find the correction area. Figure 6a shows an example of detecting the boundary of the projection surface masks $M_{s}\left(x,y\right)$. Second, we create a distortion shape image at the user’s viewpoint in order to calculate the correction area from the detected boundary pixels efficiently. To do this, we perform a process similar to a computer graphics pipeline. The boundary pixels on the depth image are transformed into 3D points in the 3D user coordinates according to Equation (5), as shown in Figure 6b. These 3D points are projected onto a plane on the viewing volume of the user’s perspective. Then, we rasterize the projected points into a 512×424 image, as shown in Figure 6c. In the rasterization step, a polygon filled with the white color is drawn by using the projected 2D points with orders provided by the contour detection method [48]. Finally, the largest rectangle inside the filled regions with the white color of the rasterized image is calculated by Vandevoorde’s method [49].

After the largest rectangle (the correction area) is found on the 2D distortion shape image, as shown in Figure 6, we now define it using the 3D user coordinates for use in the correction process. Therefore, we define the largest rectangle as the correction area using the fields of view and transformations parametrically as

(6) $$\begin{array}{cc} T_{x_{c}}=-\left(\frac{x_{c}}{r_{x}}-\frac{W_{p}}{2}\right)+\frac{w_{c}}{2},\; & T_{y_{c}}=-\left(\frac{y_{c}}{r_{y}}-\frac{H_{p}}{2}\right)+\frac{h_{c}}{2},\\ fov_{x_{c}}=2\tan^{-1}\frac{w_{c}}{2r_{x}Z_{plane}},\; & fov_{y_{c}}=2\tan^{-1}\frac{h_{c}}{2r_{y}Z_{plane}},\\ r_{x}=W_{r}/W_{p},\;r_{y}=H_{r}/H_{p}, & \end{array}$$

where $fov_{x_{c}}$, $fov_{y_{c}}$, $T_{x_{c}}$, and $T_{y_{c}}$ are the fields of view and transformations for defining the largest rectangle as the correction area in the 3D user coordinates, $\left(x_{c},y_{c},w_{c},h_{c}\right)$ represents the largest rectangle on the 2D distortion shape image relative to the starting point, width and height, respectively. $W_{p}$ and $H_{p}$ in Equation 6 are the width and height sizes of projection plane, respectively, and $W_{r}$ and $H_{r}$ are the width and height sizes of the rasterized image, respectively. In this way, we can parametrically define the rectangular correction area using the 3D user coordinates as FOVs ($fov_{x_{c}}$ and $fov_{y_{c}}$) and transformations ($T_{x_{c}}$ and $T_{y_{c}}$).

### 3.6. Warping and Correction

In our calibration, we can transform the 3D depth image coordinates into the 2D projection image coordinates by using Equation (3), but it is impossible to convert them in the opposite direction, as it is an ill-posed problem. However, the depth information (3D information) of the projection image is essential for our undistortion process. Fortunately, we can somewhat calculate the depth of the projection image using sparse 3D information via our calibration and the depth image. Therefore, we must estimate unknown parts of the depth information in the projection image.

**Figure 7:**
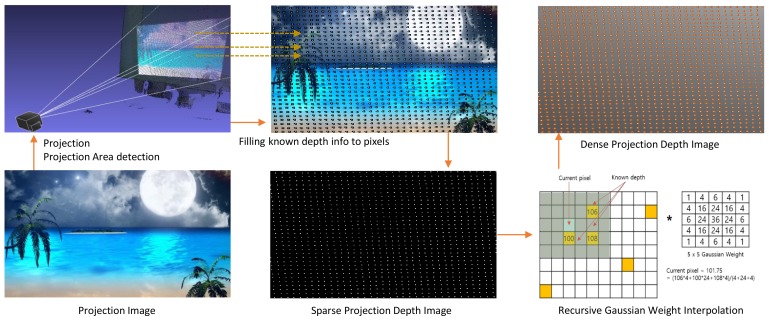
An example of generation of the dense projection depth image by recursive Gaussian weight interpolation.

Existing correction methods [7,9] re-construct 3D parametric or meshed surfaces by fitting known 3D points to estimate the unknown information. However, these approaches have high computation costs, resulting in the slow correction of the geometric distortion of the projection. Instead, we generate a sparse projection depth image from known depth information in the projection image. Then, we quickly convert a sparse projection depth image into a dense projection depth image using Gaussian weight interpolation with 5×5 masks. Figure 7 shows the whole process of generating the dense projection depth image.

For generating a warping table to correct the projection distortion, we transform the dense projection depth image into 3D point clouds as $P_{u}\left(x,y\right)=\left\{x_{u}\left(x,y\right),\;y_{u}\left(x,y\right),\;z_{u}\left(x,y\right)\right\}\in R^{3}$ in the user coordinates via intrinsic parameters of the projector and $M_{pu}$. We calculate the angles in the correction area, showing that each 3D point of $P_{u}\left(x,y\right)$ is included in the correction area parametrically as:

(7) $$\begin{array}{c} \theta_{x}\left(x,y\right)=2\tan^{-1}\left(\frac{x_{u}\left(x,y\right)-T_{x_{c}}z_{u}\left(x,y\right)Z_{plane}^{-1}}{z_{u}\left(x,y\right)}\right),\\ \theta_{y}\left(x,y\right)=2\tan^{-1}\left(\frac{y_{u}\left(x,y\right)-T_{y_{c}}z_{u}\left(x,y\right)Z_{plane}^{-1}}{z_{u}\left(x,y\right)}\right), \end{array}$$

where $Z_{plane}$ is the *z*-axis value for the perspective projection of distortion at the user’s viewpoint, $\theta_{x}\left(x,y\right)$ is the angle relative to the *x*-axis, and $\theta_{y}\left(x,y\right)$ is the angle relative to the *y*-axis. Then we check if these angles are inside the correction area as

(8) $$\begin{array}{c} \theta_{x}\left(x,y\right)\leq fov_{x_{c}},\\ \theta_{y}\left(x,y\right)\leq fov_{y_{c}}, \end{array}$$

When Equation (8) is satisfied, $P_{u}\left(x,y\right)$ is inside the correction area (the largest rectangle). Using the fields of view and transformations that define the correction area, we calculate the image positions to be displayed for correction by

(9) $$\begin{array}{cccc} x_{c}= & \frac{w\cdot \theta_{x}\left(x,y\right)}{2\cdot fov_{x_{c}}} & +\frac{w}{2} & ,\\ y_{c}= & \frac{h\cdot \theta_{y}\left(x,y\right)}{2\cdot fov_{y_{c}}} & +\frac{h}{2} & , \end{array}$$

where *w* and *h* represent the width and height of the projection image, respectively, and $\left(x_{c},y_{c}\right)$ is the position of the pixel to be displayed at (x,y). Finally, we make a warped projection image to correct the distortion given by

(10) $$\begin{array}{c} g\left(x,y\right)=f\left(x_{c},y_{c}\right), \end{array}$$

where *g* is the warped image and *f* represents the original projection image. This process can warp the projection image through backward mapping, thus constructing the corrected image quickly.

## 4. Result and Analysis

To investigate the effectiveness of the proposed calibration and correction methods on our DIW system, we conducted both quantitative and qualitative experiments under various conditions. The depth and RGB cameras are built in Kinect v2 with 512×424 and 1920×1080 resolutions, respectively. For testing, the projector and Kinect v2 were connected to a laptop (MacBook Pro 2015 early with i5 2.7 GHz and 8 GB RAM) on which we ran our DIW system with the proposed calibration and correction methods. Since the depth image produced by Kinect v2 has lens distortion, we preprocessed correction of the lens distortion using Brown’s distortion model [50] with 5 correction parameters, $K_{1}$, $K_{2}$, $K_{3}$, $P_{1}$, and $P_{2}$, which are supplied by the manufacturer, as shown in Table 1. As our laser projector does not use a lens for projection [51], we did not consider lens distortion in our tests. For user position, we set up $H_{pu}$ by assuming the user is located one meter behind the projector for all experiments.

### 4.1. Quantitative Evaluations

**Figure 8:**
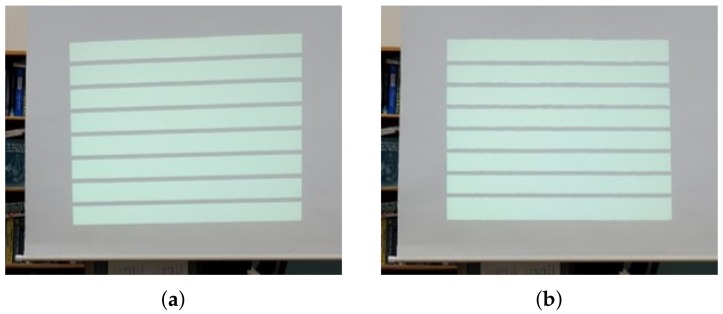
Test results of the light straight ratio errors, which are captured using the camera at the user position. (**a**) The distorted screen. (**b**) The undistorted screen.

We first performed a comparative evaluation with the methods in [14,21] to determine the reprojection accuracy of the proposed calibration method. We also tested the correction accuracy as we projected and corrected horizontal/vertical line images in Figure 8 and measured the straightness of the lines on the corrected image. Finally, to verify the speed of the distortion correction method, which is essential to our DIW system, we performed a comparison evaluation for the correction time of each image with the methods in [7,9].

#### 4.1.1. Reprojection Errors of Calibration

Our calibration method has the advantages that it can be applied directly to detect the projection surface and cope with variable intrinsic parameters such as screen resolutions in the projector. Thus, we tested the calibration accuracy of our method while it keeps these advantages. We calculated the reprojection errors of our method. Then, we compared our calibration method with the existing calibration methods in [14,21], which use an RGB camera and depth camera for calibration, respectively. For comparison, all methods being tested for calibration accuracy use the same number of samples, which are obtained by projecting a 9×6 chessboard to 25 different surfaces (i.e., a total of 1350 samples under test).

**Figure 9:**
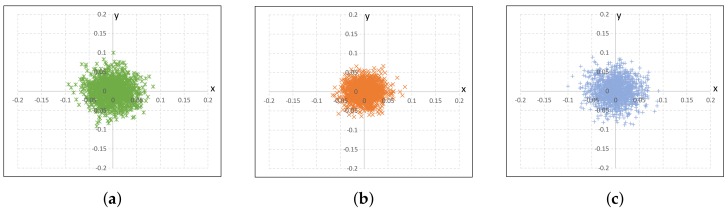
2D histograms of the re-projection errors, which are calculated with respect to the projector image coordinates: (**a**) the RGB camera based method (std = 0.027), (**b**) the depth camera based method (std = 0.029), (**c**) the proposed method (std = 0.022).

Table 2 shows the calibration results of our proposed method and the existing ones. In addition, we calculated the reprojection errors of the three methods, as shown in Figure 9. These results show that all three methods have similar results. However, our calibration is specialized to detect the projection surface directly and quickly for DIW, and it can easily cope with projector resolution changes.

#### 4.1.2. Line Straightness Error Ratio Test

**Figure 10:**
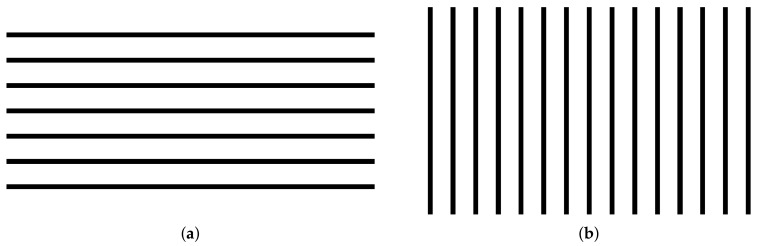
The line images for the line straightness error ratio test. (**a**) The lines image with seven horizontal lines. (**b**) The lines image with fifteen vertical lines.

In order to test precision of the proposed correction method quantitatively, we calculated the straightness of vertical and horizontal line images, which are captured at the user position using a camera. To calculate the simple gradients of lines on the line images, we propose using the Line Straightness Error Ratio (LSER) given by:

(11) $$\begin{array}{cc} LSER_{v}=1-\left|\frac{\tan(90{}^{\circ}-k)}{\tan(T_{e})}\right|,\; & LSER_{h}=1-\left|\frac{\tan(k)}{\tan(T_{e})}\right|, \end{array}$$

where $LSER_{v}$ is the line straightness error ratio for vertical straight lines, $LSER_{h}$ is the line straightness error ratio for the horizontal lines, *k* is the angle with respect to the image *x*-axis of the image, and $T_{e}$ is the tolerance degree allowed by the error ratio. In this study, we set $T_{e}$ as 10°, which means that LSER is 1 with no distortion (k=0) and LSER is 0 with k=10°. If the angle *k* is larger than the tolerance degree $T_{e}$, it has a negative value. To measure LSER, the line pattern images shown in Figure 10 are projected and corrected by the proposed method. Then, we captured them using the camera, and *k* is calculated with two edge points at the end of each line. We repeated the LSER experiments five times in horizontal and vertical images, respectively, for the accuracy. Table 3 and Figure 8 show our LSER experimental results. Based on these results, our correction method has a good LSER performance over 0.99 at $M_{e}=10{}^{\circ}$. This means that our correction results have a less than 0.1° difference in the straight lines, indicating that the proposed method can accurately correct distortion on non-planar surfaces.

#### 4.1.3. Performance Test for Running Speed

In the proposed DIW, fast correction is considered an important requirement since the distortion of a dynamic non-planar surface need to be estimated and corrected in real time. Therefore, we compared the correction speed of the proposed method with the conventional RGB camera-based methods. To test the performance of the correction speed, we measured the time needed to correct an image with a 1920×720 resolution, which is the maximum resolution of our projector. Table 4 shows the correction times of the proposed and existing methods, indicating that the proposed method can correct the distortion of the image very quickly compared to the conventional methods. The main reason for the fast correction of our method is that we use the depth camera to predict the distorted surface directly, followed by filling in the missing information using simple interpolation. This experiment shows that the fast correction of our method is appropriate for dynamic surfaces, which is essential for DIW.

### 4.2. Qualitative Evaluations

In this paper, we carried out not only quantitative evaluation, but also qualitative evaluation for the proposed DIW. For the qualitative test, we setup two scenarios in which our DIW system can be used in practice: (i) we built a large screen using UAVs and a lightweight canvas to evaluate the practical use of our DIW system in space-constrained environments such as celebrations; and (ii) we manually made dynamic non-planar surface to evaluate extreme environments.

**Figure 11:**
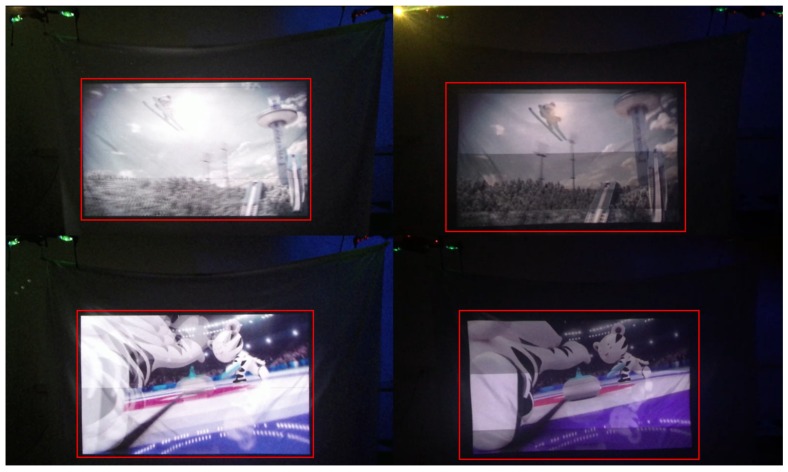
DIW results from dynamic non-planar surfaces generated from UAVs and a lightweight canvas. The left column depicts the result obtained with the proposed method and the right column shows the output of a conventional projector.

**Figure 12:**
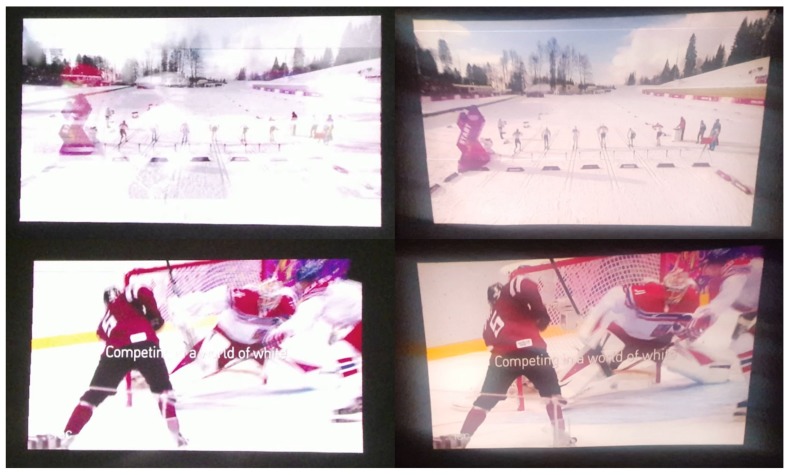
DIW results from dynamic non-planar surfaces manually generated by a human. The left and right columns depict the result obtained with the proposed method and the output of a conventional projector, respectively.

Figure 11 and Figure 12 show the qualitative results of our method, where the left column in each figure depicts the result obtained with the proposed method and the right column shows the output of a conventional projector. Our results show that the proposed DIW system effectively corrects distortion, resulting in a TV-like rectangular screen. Furthermore, our method shows good undistortion results on the dynamic non-planar surfaces made from UAVs and lightweight canvas. These results demonstrate that our correction method and DIW system can be useful in real and extreme environments.

To show the effectiveness of our DIW system, we uploaded a video clip (**Video 1**) at “https://youtu.be/RIpj0pED6NA”. The first part of **Video 1** demonstrates the performance of our DIW system that consists of UAVs and a lightweight canvas. The second part of **Video 1** shows the performance of our DIW system in an extreme case where screen geometries severely change in time. In **Video 1**, the left part of the screen shows projection images generated by our DIW system, and the right part of the screen shows projection images displayed by a projector only.

As shown in **Video 1**, screens with UAVs and canvas have irregular local and global movements due to wind of UAVs. In this case, our DIW system stably produces undistorted square screens in real time. On the other hand, the projection-only system yields clearly noticeable distortion. Regarding the second scenario, the projector-only system causes significant geometric distortions on extremely varying screen surfaces, while the proposed DIW system consistently provides rectangular-shaped images on that surface.

## 5. Limitations

Our DIW system uses a projector, which is fundamentally difficult to use in bright environments, especially during the daytime under outdoor conditions. This problem is a fundamental limitation of our DIW system. In recent years, off-axis projectors have been introduced that generate projection screens in a slightly upward direction to create large screens at a short distance. Regarding off-axis projectors, our DIW system cannot be applied to them, as off-axis projectors require different calibration and correction formulations than the proposed methods. Finally, in our method, the user position is manually set for use in the user-perspective distortion correction. This means that if the user’s position changes continually, our DIW system cannot track the user’s position and thus does not generate perceptually correct screens. This problem remains for future work.

## 6. Conclusions

In this paper, we propose a new display system, namely “Display in the Wild”, to overcome limitations of the conventional displays. To implement DIW, we suggest both hardware and software configurations. For the hardware of DIW, we select the projector and depth camera in order to generate screen anywhere with different sizes and to correct the geometric distortion occurring on dynamic non-planar surfaces in real time. For the software configuration, we propose a DIW-specific calibration between the projector and depth camera. In addition, we also propose a fast correction method that correct the geometric distortions on dynamic and non-planar surfaces. Our experimental results prove that the proposed DIW system generates the rarely distorted screen on a dynamic non-planar surface in real time, thus showing usefulness in practical scenarios (e.g., lightweight screen using UAVs) and under extremely varying surface conditions. Since the proposed method can be divided into per-pixel processing, we consider extending our DIW system to ultra-high definition (UHD)-based projector applications via implementing parallel processing with GPUs for future work.

## Figures and Tables

**Table 1:** Correction parameters of the lens distortion for Brown’s distortion model on Kinect v2 provided by the manufacture.

Correction Parameters	Value
$K_{1}$	0.09558
$K_{2}$	−0.26799
$K_{3}$	0.08755
$P_{1}$	0.00000
$P_{2}$	0.00000

**Table 2:** Calibration results of the proposed method and existing methods.

Method	$R_{dP}$	$T_{dP}$
The proposed method	$\begin{array}{ccc} 0.8856 & -0.0116 & -0.0109\\ 0.0102 & 0.8583 & 0.0072\\ 0.0091 & -0.0142 & 0.9998 \end{array}$	$\begin{array}{c} 16.7414\\ 17.2027\\ -278.4640 \end{array}$
The method [14]	$\begin{array}{ccc} 0.8801 & -0.0123 & -0.0138\\ 0.0135 & 0.8553 & 0.0076\\ 0.0059 & -0.0101 & 0.9967 \end{array}$	$\begin{array}{c} 16.7350\\ 17.1968\\ -278.4688 \end{array}$
The method [21]	$\begin{array}{ccc} 0.8871 & -0.0083 & -0.0070\\ 0.0151 & 0.8595 & 0.0174\\ 0.0152 & -0.0088 & 1.0050 \end{array}$	$\begin{array}{c} 16.7446\\ 17.2056\\ -278.4625 \end{array}$

**Table 3:** Line Straightness Error Ratio results of the proposed correction method.

Test Number	Vertical Lines	Horizontal Lines
1	0.99210	1.00000
2	0.99209	0.99111
3	0.99605	0.99111
4	0.99210	0.99115
5	0.98157	1.00000
Average	0.99078	0.99467

**Table 4:** The correction time (seconds) results of the proposed method and existing methods.

Method	Projection Surface Prediction	Correction	Total
The proposed method	0.012	0.091	0.103
The method in [7]	4.324	5.421	9.745
The method in [9]	3.875	4.273	8.148

## Appendix A

Dividing the numerator and denominator of the right-hand side of Equation (3) by $r_{33}$ and replacing $r_{11}/r_{33},r_{12}/r_{33},\cdots ,t_{3}/r_{33}$ with $l_{1},l_{2},\cdots ,l_{11}$, respectively, yield:

(A1) $$\begin{array}{cc} m_{1}z_{d}= & l_{1}x_{d}+l_{2}y_{d}+l_{3}z_{d}+l_{4}-m_{1}l_{9}x_{d}-m_{1}l_{10}y_{d}-m_{1}l_{11},\\ m_{2}z_{d}= & l_{5}x_{d}+l_{6}y_{d}+l_{7}z_{d}+l_{8}-m_{2}l_{9}x_{d}-m_{2}l_{10}y_{d}-m_{2}l_{11}, \end{array}$$

where $m_{1}=x_{i}f_{px}^{-1}$, $m_{2}=y_{i}f_{py}^{-1}$. To solve Equation (A1), we set $l_{1}\;∼\;l_{11}$ as the unknown calibration parameters and produce a system of linear equations Ax=b as follows:

(A2) $$\begin{array}{c} {\left[\begin{array}{ccccc} x_{d_{i}} & 0 & \cdots & x_{d_{N}} & 0\\ y_{d_{i}} & 0 & \cdots & y_{d_{N}} & 0\\ z_{d_{i}} & 0 & \cdots & z_{d_{N}} & 0\\ 1 & 0 & \cdots & 1 & 0\\ 0 & x_{d_{i}} & \cdots & 0 & x_{d_{N}}\\ 0 & y_{d_{i}} & \cdots & 0 & y_{d_{N}}\\ 0 & z_{d_{i}} & \cdots & 0 & z_{d_{N}}\\ 0 & 1 & \cdots & 0 & 1\\ -m_{1_{i}}x_{d_{i}} & -m_{2_{i}}x_{d_{i}} & \cdots & -m_{1_{N}}x_{d_{N}} & -m_{2_{N}}x_{d_{N}}\\ -m_{1_{i}}y_{d_{i}} & -m_{2_{i}}y_{d_{i}} & \cdots & -m_{1_{N}}y_{d_{N}} & -m_{2_{N}}y_{d_{N}}\\ -m_{1_{i}} & -m_{2_{i}} & \cdots & -m_{1_{N}} & -m_{2_{N}} \end{array}\right]}^{T}\left[\begin{array}{c} l_{1}\\ l_{2}\\ l_{3}\\ l_{4}\\ l_{5}\\ l_{6}\\ l_{7}\\ l_{8}\\ l_{9}\\ l_{10}\\ l_{11} \end{array}\right]\;=\;\left[\begin{array}{c} m_{1_{i}}z_{d_{i}}\\ m_{2_{i}}z_{d_{i}}\\ \vdots \\ m_{1_{N}}z_{d_{N}}\\ m_{2_{N}}z_{d_{N}} \end{array}\right], \end{array}$$

where *N* is the number of points in our linear system, $\left(x_{d_{i}},y_{d_{i}},z_{d_{i}}\right)$ is *i*-th 3D point in the depth camera coordinate, and $\left(m_{1_{i}},m_{2_{i}}\right)$ is the respective inverse product of the *i*-th 2D image point and the focal length of the projector. We can solve Equation (5) if we know at least eleven pairs of 3D points in the depth camera coordinates with the corresponding 2D points in the image coordinates.

We divide the all elements in the numerator and denominator of the right-hand side in Equation (A1) by $r_{33}$. In order to avoid a homogeneous system of linear equation with b=0. Note that we specifically choose $r_{33}$ as the divisor since $r_{33}\left(=\cos\left(\theta_{x}\right)\cos\left(\theta_{y}\right),\mathrm{where}\;0\leq \theta_{x},\theta_{y}\ll \frac{\pi }{2}\right)$ is often greater than zero, thus avoiding unstable results when the denominator is close to zero. Another advantage of choosing $r_{33}$ as the divisor is that we can easily recover $r_{33}$ and $H_{dp}$ from the calibration parameters. Using $l_{10}$, we calculate $\theta_{x}$ as follows:

(A3) $$\begin{array}{c} l_{10}=\frac{r_{32}}{r_{33}}=\frac{-\sin\left(\theta_{x}\right)\cos\left(\theta_{y}\right)}{\cos\left(\theta_{x}\right)\cos\left(\theta_{y}\right)}=-\tan\left(\theta_{x}\right) \end{array}$$

Using $l_{9}$ and $\theta_{x}$, we calculate $\theta_{y}$ as follows:

(A4) $$\begin{array}{c} l_{9}=\frac{r_{31}}{r_{33}}=\frac{-\sin(\theta_{y})}{\cos\left(\theta_{x}\right)\cos\left(\theta_{y}\right)}=-\frac{\tan(\theta_{y})}{\cos(\theta_{x})}, \end{array}$$

Then, we calculate $r_{33}$ by using $\theta_{x}$ and $\theta_{y}$, which allows us to recover the transformation matrix $H_{dp}$ from $l_{1}\;∼\;l_{11}$.
